# Quantum Medicine and Irritable Bowel Syndrome-Associated Chronic Low-Back Pain: A Pilot Observational Study on the Clinical and Bio-Psycho-Social Effects of Bioresonance Therapy

**DOI:** 10.3390/medicina60071099

**Published:** 2024-07-05

**Authors:** Giovanni Barassi, Giuseppe Alessandro Pirozzi, Angelo Di Iorio, Raffaello Pellegrino, Piero Galasso, Dietmar Heimes, Barbara Praitano, Pier Enrico Gallenga, Loris Prosperi, Antonio Moccia, Maurizio Panunzio

**Affiliations:** 1 Center for Physiotherapy, Rehabilitation and Re-Education (Ce.Fi.R.R.), Venue “G. d’Annunzio” University of Chieti-Pescara, 66100 Chieti, Italy; pierogalassomail@gmail.com (P.G.); heimes.cefirr@gmail.com (D.H.); gallengape@gmail.com (P.E.G.); lorispro90@gmail.com (L.P.); fisiomoccia.a@gmail.com (A.M.); 2 Responsible Research Hospital, 86100 Campobasso, Italy; giuseppealessandro.pirozzi@responsible.hospital (G.A.P.); m.panunzio@responsiblecapital.ch (M.P.); 3 Department of Innovative Technologies in Medicine & Dentistry, “G. d’Annunzio” University of Chieti-Pescara, 66100 Chieti, Italy; angelo.diiorio@unich.it; 4 Department of Scientific Research, Campus Ludes, Off-Campus Semmelweis University, 6912 Lugano, Switzerland; raffaello.pellegrino@ucm.edu.mt; 5 Faculty of Medicine and Surgery, Degree Course in Physiotherapy, “Catholic University” of Rome/Campobasso, 86100 Campobasso, Italy; barbara.praitano@responsible.hospital

**Keywords:** irritable bowel syndrome, low-back pain, quality of life, Bioresonance Therapy, Calprotectin

## Abstract

*Background and Objectives*: Irritable bowel syndrome (IBS) is an invasive and potentially disabling syndrome characterized by a multitude of symptoms capable of reducing the quality of life of patients. Among the most disabling symptoms of IBS is certainly physical pain, which manifests itself mainly at the abdominal level but can also appear in other areas of the body, particularly in the form of chronic low-back pain (CLBP). Among the non-invasive methods of treating organ-specific pathologies and organ-related musculoskeletal problems, the use of Bioresonance Therapy (BT)—based on the administration of self-modulating Extremely Low-Frequency Electromagnetic Fields, capable of determining a rebalance of bio-electrical and metabolic activity in the presence of various functional alterations—is currently gaining acceptance. Therefore, we decided to monitor results obtained from patients suffering from IBS and CLBP subjected to a cycle of treatments with BT. *Materials and Methods*: We monitored 20 patients (12 women and 8 men, average age of 51 years) suffering from CLBP and other visceral symptoms related to IBS. Patients were monitored through the use of the Bristol Stool Form Scale (BSFS), the Fecal Calprotectin test and the Short-Form Health Survey 36 (SF-36), collected before (T0) and after (T1) the execution of the cycle of treatments. They undertook a treatment protocol consisting of eight sessions of BT carried out over about a month. *Results*: At the end of the treatments with BT, it was possible to observe a general and significant improvement in all the parameters observed, as well as a close inversely proportional correlation between the Calprotectin values detected and the quality of life experienced by the patients in relation to their perceived IBS symptoms. *Conclusions*: Overall, our pilot study would seem to suggest a potential beneficial effect of BT in modulating organic and musculoskeletal symptoms derived from IBS.

## 1. Introduction

Irritable bowel syndrome (IBS) is an invasive pathology of the digestive system that, according to statistics from the World Gastroenterology Organization, affects a percentage varying from 3.8% to 23% of the entire world population, referring to the various diagnostic criteria formulated by the ROME Foundation [1,2,3]. The percentage of IBS patients in search of treatment reaches 12% in primary care practice, representing the largest subgroup seen in gastroenterology clinics [1]. IBS is complex in terms of both etiopathogenesis and symptoms. It is usually classified into subgroups: IBS with constipation (IBS-C), IBS with diarrhea (IBS-D) and mixed IBS (IBS-M) [1].

Typically, IBS diagnosis is based on the observation of symptoms according to the Rome IV Criteria [4]: IBS is diagnosed when a patient presents recurrent abdominal pain on average at least 1 day/week in the last 3 months, with the first onset of symptoms dating back to at least 6 months before the first specialist visit [4]. This pain should be present during defecation or in association with a change in frequency or appearance of stool [4].

Although various mechanisms have been suggested for IBS, close neurobiological correlations underlying its development have been highlighted. In fact, the so-called “brain–gut microbiome” model is being increasingly investigated, exposing the existence of a complex series of physiological connections related to the development of IBS symptoms [5]. These neurophysiological connections develop along an organized and bidirectional communicational axis between the central nervous system and the digestive system; this axis enables an informational and operational exchange between intestinal functioning and the emotional and cognitive state of the body [6].

IBS symptoms can take on a visceral, somatic or neurological characterization, ranging from abdominal pain and cramps to anxiety and depression, sometimes invading even organs distant from the digestive system [7]. Moreover, pain may appear or radiate to areas adjacent to or distant from the abdominal one [5].

Among the symptoms, there is a high prevalence of chronic low-back pain (CLBP) in patients with IBS, reaching a percentage level of 29–37.3%, well above the 3.9–20.3% prevalence in the general population [8]. The severity of CLBP in IBS patients may seriously compromise their quality of life, making them 50% more likely to resort to spinal surgery, which often proves to be an unsuccessful and counterproductive strategy [9]. CLBP occurs significantly more in patients with IBS compared with patients with other pathologies of the digestive system, including inflammatory bowel disease (IBD) [10]. Therefore, functional gastrointestinal disorders (FGIDs), such as IBS, take a prominent place within the “functional somatic syndromes’’, together with chronic fatigue syndrome and fibromyalgia, with which they frequently overlap [11].

IBS can lead to different physiological-organic alterations, allowing to highlight some key aspects of its diagnosis and monitoring.

The first thing to check in the presence of IBS is the defecation quality, referring to the Bristol Stool Form Scale (BSFS). The BSFS is based on the observation of the shape and consistency of the feces. Typically, it enables the identification of constipation (grades 1 and 2), healthy defecation (grades 3 and 4) and a tendency toward fecal incontinence (grades 5, 6 and 7) [12]. The BSFS appears to be effective for identifying subforms of IBS such as IBS-C (grades 1 and 2) and IBS-D (grades 5, 6 and 7) according to the Rome IV Criteria [4].

A second factor to consider is Fecal Calprotectin (FC), which is a 36 kDa member of the S100 protein family derived from neutrophils and has direct antimicrobial and immune effects [13]. Calprotectin levels are proportional to the degree of any existing inflammation, and its concentration in feces is about six times that of plasma [13]. It is used to highlight intestinal inflammation, with values that typically exceed 50 mcg/g in the presence of intestinal inflammation (such as IBD) or intestinal dysmotility (as in the case of IBS) [14]. In fact, it seems that, in IBS, especially of the IBS-D type, there may still be a sub-clinical inflammatory state associated with the greater permeability of the intestinal membrane, leading to an increase in FC values [14].

Given the complexity of IBS, it is important to identify the most effective and minimally invasive therapies to improve the health of patients. In this regard, alternative and complementary therapies typical of other healthcare fields, such as, for example, rehabilitation, may also be useful.

One of the most innovative approaches in the field of somato-visceral disorders is that of Quantum Medicine (QM) [15,16,17,18,19]. This medical–health branch, which is spreading in the rehabilitation field, is based on the application of electromagnetic stimuli consistent with the electromagnetic activity of the human body, exploiting Extremely Low-Frequency Electromagnetic Fields (ELF-MFs), also known as Bioresonance Therapy (BT), to determine a rebalancing of the metabolic–neurophysiological activities of the organism and to counteract various pathological states [19]. The therapeutic effects of QM techniques seem to depend on different pathways. QM techniques are capable of counteracting oxidative stress, reducing levels of inflammation and increasing cellular metabolism and the production of mitochondrial ATP [15,16,17,18,19]. This therapeutic approach seems to be effective even for localized musculoskeletal disorders such as low-back pain [20].

Technically, BT is based on the concept that organic processes emit electromagnetic oscillations of different frequencies, intensities, durations and waveforms. These oscillations, which are also the basis of Bioresonance Diagnostics, appear to be organ-specific and pathology-specific [21,22,23,24]. BT uses ELF-MFs capable of functionally interfering with the altered frequencies in the body of the patient [25]. Specifically, Paul Schmidt developed a therapeutic system based on a 152 kHz carrier frequency, which is then modulated according to the feedback deriving from the electromagnetic field of the patient [26].

Therefore, based on these considerations, we carried out a pilot observational study to investigate the therapeutic potential of BT in the presence of IBS associated with CLBP.

## 2. Materials and Methods

This research was a pilot retrospective analytical observational study carried out at the Gemelli Molise Hospital (Campobasso, Italy) cooperating with Ce.Fi.R.R. (Center for Physiotherapy, Rehabilitation and Re-education) staff from November to August 2023.

The rehabilitation protocol observed is safe, as all the procedures applied comply with the safety regulations of the country where the study was made; the protocol is accessible to anyone who does not highlight specific contraindications during the initial clinical evaluation necessary for all patients who access the hospital; the only major contraindications to access the protocol are pregnancy, epilepsy, electrical implants, tumors, infections, tuberculosis and serious heart disease. The protocol does not constitute an experimental practice, as it applies the same procedures used for all patients who do not present the aforementioned contraindications. This study was conducted in accordance with the ethical principles of the Declaration of Helsinki. Written informed consent was obtained at enrolment from participants who were willing and able. Due to these considerations and the lack of incontrovertible national legislation regarding the need for the submission of retrospective and/or non-pharmacological observational studies to an ethics committee [27], normal ethics committee clearance was not required [28].

A total of 20 patients (12 women and 8 men; Caucasian ethnicity; average age of 51 years) were observed within the Gemelli Molise Hospital (Campobasso, Italy).

All patients turned to hospital specialists due to the presence of frequent episodes of abdominal pain accompanied by CLBP, which intensified mainly during defecation, with temporal criteria consistent with that of Rome IV for the diagnosis of IBS [4].

To assess the status of IBS before (T0) and after (T1) the therapeutic protocol, an evaluation of the patients was carried out using 3 validated diagnostic tools:

- BSFS: The evaluation of the appearance of the stool was carried out by the doctors evaluating the patients observed through careful visual inspection of a stool sample collected at times T0 and T1.
- FC: A Calprotectin assay was carried out on the same stool samples, collected at times T0 and T1, and used for the BSFS evaluation. The analysis of the samples was carried out at the Gemelli Molise Hospital (Campobasso, Italy) reference laboratories using an immunochromatographic kit with a densitometric reader for quantitative dosing, called CalFast XT (Eurospital S.p.A., Trieste, Italy).
- Short-Form Health Survey 36 (SF-36): This is a questionnaire typically used to evaluate the general state of health of a patient, with particular reference to the physical and social limitations determined by their pathology. It consists of 36 questions divided into 8 subclasses: physical functioning (10 items), limitations due to physical health (4 items), limitations due to emotional problems (3 items), energy and fatigue (4 items), emotional well-being (5 items), social activities (2 items), pain (2 items), general health perception (5 items) and general health status variability in the last year (1 item) [29]. Various studies have highlighted how patients suffering from IBS have lower SF-36 scores than the average, not only in relation to the physical limitations of the pathology but also due to its psycho-emotional and social implications [30]. The questionnaire was administered to the patients at times T0 and T1.

**Figure 1:**
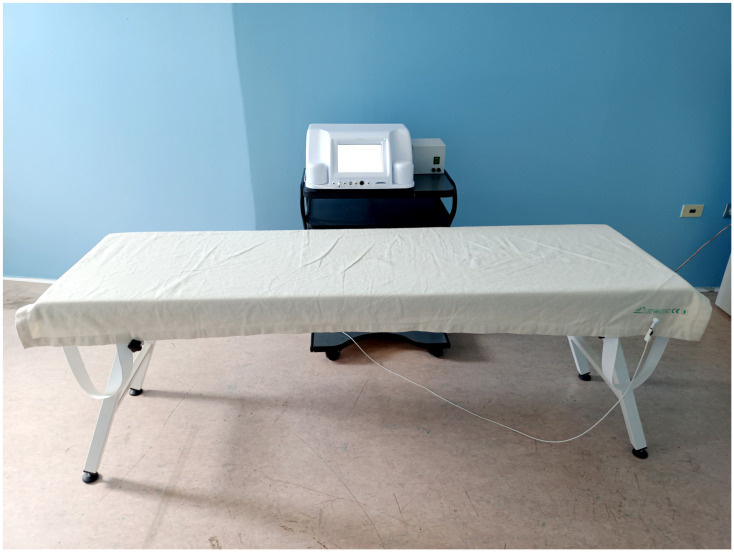
The Rayocomp PS 1000 Polar 4.0 Med BT device used to treat the observed patients.

In order to intervene in the diagnosed IBS, in agreement with the reference medical evaluators in undertaking an alternative therapeutic path, patients underwent treatment through BT. For this study, the BT device used was the Rayocomp PS 1000 Polar 4.0 Med (Rayonex Biomedical GmbH, Lennestadt, Germany). It is composed of an ELF-MF generator that transmits a magnetic field to a fabric mattress containing the emitting elements (Figure 1). The ELF-MFs of the device are imperceptible to the patient, producing no visual, auditory, thermal or tactile sensations.

The patient is made to lie on the BT mattress, positioned on a common physiotherapy table, in a supine and relaxed position, wearing underwear and deprived of all personal removable metal or electrical elements (jewelry, glasses, mobile phone, etc.), so as to limit any possible electromagnetic disturbance for the BT. The specific treatment programs for the pathology addressed are selected through the software of the ELF-MFs generator connected to the mattress, consistently with the protocols derived from the experiences of Paul Schmidt [26]. Each session involved the application of a program specifically designed for IBS, composed of the 12 phases indicated in Table 1, with each session lasting 65 min. Each patient underwent 1 session every 48 h for approximately 1 month, until completing a total of 8 sessions.

Data are reported as absolute number and frequency. Linear mixed models, with random intercept and random slope, were applied to assess the potential association between the times of the study and Calprotectin. Lastly, to assess variation in the SF-36 score and in every subscale, generalized estimating equations (GEEs) were applied with the Poisson option.

SAS 9.4 for Windows (SAS Institute Inc., Cary, NC, USA) was used for all data processing. We set the level of statistical significance at *p* < 0.05 (2-sided).

## 3. Results

At baseline, according to BSFS classification, 3 patients (15%) were classified as constipated (grade 1 or 2, IBS-C); 5 patients (25.0%) showed a normal fecal quality (grade 3 or 4); and 12 patients (60.0%) presented inconsistent fecal formation (grade 5 to 7, IBS-D). At the end of the study, only 1 (5%) subject reported constipation, while 5 patients (25.0%) had inconsistent fecal formation, and the remaining 14 subjects (70%) presented normal fecal formation. These results indicate a tendency toward normalization of fecal quality in patients treated with BT, especially those with an inconsistent fecal formation at time T0.

To assess whether a correlation between FC changes and the time of the study could be found, we used the linear mixed model approach. Independently from the time of the study, in the whole population, the mean value for FC was 62.76 ± 9.25; the estimated-ρ was equal to 33.3%, meaning that almost one-third of the total variance was attributable to between-subject differences. When the differences between times are considered in the model, the mean value for FC is statistically significantly higher at baseline (β ± SE: 34.27 ± 8.21; *p*-value < 0.001) compared with the follow-up. The comparison between the two models demonstrates that about 46.5% of the variance between subjects can be explained by time, meaning that the intervention halved the mean value of FC. The change in the BSFS score, when included in the model, is not statistically significant and does not change the strength of the association between the times of the study and Calprotectin.

**Figure 2:**
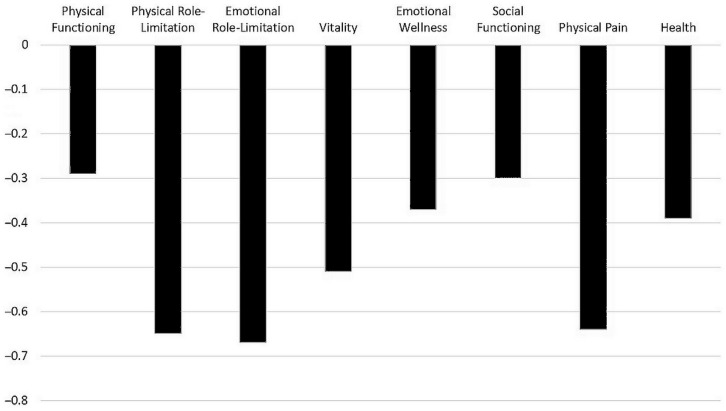
GEE estimates (β ± SE) for the comparison between times of the study for the items of the SF-36.

Figure 2 reports the generalized estimating equation (GEE) estimates (β ± SE) for the comparison between times of the study (where follow-up is the reference time) for the items of the SF-36; all the models were adjusted for Calprotectin levels (Supplementary Material Table S1). Physical-role-limitation (−0.65 ± 0.22; *p*-value = 0.004), emotional-role-limitation (−0.68 ± 0.24; *p*-value = 0.006), vitality (−0.51 ± 0.11; *p*-value < 0.001) and physical-pain (−0.64 ± 0.13; *p*-value < 0.001) improved by at least 50% from baseline to the end of the study; all the other SF-36 items also significantly improved but to a lesser extent. Calprotectin was independently and statistically significant directly associated with physical pain (0.003 ± 0.001; *p*-value = 0.03) and vitality (0.002 ± 0.0001; *p*-value = 0.01); no second-order effect (time for Calprotectin) could be demonstrated (meaning that Calprotectin variation is directly associated with pain and vitality, independently of time).

## 4. Discussion

The observation of the data we collected in a small population of IBS + CLBP patients highlights how the BT treatment significantly improved both the physical (BSFS and Calprotectin) and bio-psycho-social (SF-36) parameters under observation. This result, although preliminary, represents progress in the management of IBS.

IBS has a complex and multifactorial etiopathogenesis [31]. Several mechanisms have been suggested as possible contributing causes of the pathology, identifying factors ranging from neuroendocrine alterations of the hypothalamic–pituitary–adrenal (HPA) axis [32] to alteration of the intestinal microbiota [31,33], subclinical intestinal inflammation [32,34] and modification of the body’s immune response [31,35].

Given the complexity of IBS, it is difficult to define a targeted therapeutic approach, which, to date, is mainly based on the correction of diet and lifestyle, as well as on the administration of drugs only in certain specific cases [36,37]. Therefore, the identification of new non-invasive and easy-to-apply therapies for the treatment of such a complex syndrome may be useful.

Recently, the world of mechanical–physical medicine has started to show interest in new frontiers, such as QM. This new and mostly unexplored medical specialty is based on innovative discoveries regarding the physics of water, the coherence of cellular interactions inside tissues and the interaction of ultra-weak magnetic fields with the ionic systems of the cells themselves [38,39]. Human cellular radiations are weak, but they are still also messengers, elements of cohesion and coordinators of the body’s systems, in accordance with the definition of Biofields [40]. This property is called “coherence” [41] and defines a complex network of functional connections inside living beings [42].

Since these mechanisms are based on electromagnetic interactions, BT was developed to modulate the cellular activity and state of health of the body through functional–corrective ELF-MFs. In fact, several studies have highlighted how BT can produce positive effects in the human body, such as reduction in inflammation, immune modulation and increased regeneration [15]. Such effects occur in multiple systems of the body, including the intestines [26] and the musculoskeletal system [20].

Our pilot study highlights how the fecal quality of our patients showed a generalized improvement; in fact, at time T1, 70% of the patients were in the normal range according to the BSFS (grades 3–4) [12].

Furthermore, this study highlights a significant improvement in FC values of the observed patients treated with BT. These findings agree with what has already been highlighted by some studies regarding the effectiveness of BT in the treatment of intestinal problems [26,43]. This improvement could be attributable to the ability of BT to interact with the inflammatory processes underway in the target tissues, probably through an interleukin-dependent mechanism [44].

The observed improvement in FC values faithfully follows the trend of the quality-of-life values highlighted by patients using SF-36 subscales. In fact, all SF-36 values showed a significant improvement in relation to physical-role-limitation, emotional-role-limitation, vitality and physical-pain, demonstrating a close correlation between intestinal inflammation and the patient’s quality of life. In this regard, other studies have highlighted the connection between FC levels and the quality of life of some subgroups of patients suffering from intestinal pathologies [45,46]. According to our findings, FC appears to be a strong predictor of symptoms capable of worsening the quality of life of IBS patients. Furthermore, the levels of intestinal inflammation detected on average in IBS patients observed through Calprotectin dosage would suggest that, even in the absence of a clear inflammatory component typical of IBD (with a cut-off variably indicated in the literature from 50 mcg/g to over 120 mcg/g [47,48]), in the presence of IBS, there may still be a level of subclinical inflammation capable of worsening and perpetuating its symptoms [14].

Among all the SF-36 parameters, the fact that the patients’ physical pain was also reduced, in terms of both abdominal pain and musculoskeletal pain, especially as CLBP, is particularly relevant. Considering the intrusiveness of CLBP in the lives of individuals with IBS [8,9], the fact that BT reduced their perceived pain appears interesting and worthy of attention.

Importantly, no adverse events or side effects related to the device, treatment or protocol were reported in the study.

Regarding this pilot study, it is important to underline some limitations. Firstly, given its observational retrospective nature, it was impossible to select a large and homogeneous sample, especially in terms of gender distribution. The small sample size, despite the fact that the observed patients came from a hospital with an intrinsically high number of general users, derives from the fact that BT, like other instrumental therapies, to date, still is a complementary therapy for IBS and/or CLBP and other disorders, rather than a first-line treatment, such as pharmacological, nutritional or surgical interventions [49,50]. Therefore, to date, the number of patients who, in agreement with their specialist doctors, undertake a path of this type, is still rather limited compared with the total population affected by IBS. Indeed, most of the patients undertook a conventional treatment path, which makes it difficult to observe a large number of individuals treated with an alternative approach outside of a dedicated experimental setting. Furthermore, due to time and data collection reasons, it was not possible to plan the presence of a control group or a follow-up.

Any future studies on this topic should be designed from the start as RCTs with follow-ups and a bigger and better-distributed sample to bypass these limitations. It may be particularly useful to use stratified sampling based on sex, age and IBS subtype. Furthermore, we suggest using a control group subjected to a sham treatment with the BT device turned off in order to verify the influence of the placebo effect on the results. In fact, we cannot rule out the possibility that the beneficial effects of BT in our study may be related to casual elements, so future studies are needed to prove our hypothesis right or wrong. The results obtained in this pilot observational study are encouraging, at least in terms of the short-term efficacy of BT for IBS with CLBP, although data should be analyzed with caution.

In addition to the suggested strategies for containing the limitations identified, it is necessary to produce new studies on the topic to also clarify how BT effects may vary depending on specific exposure parameters or targeted cell types, as well as underlying pathological mechanisms [51]. In this regard, it could be useful to resort to extensive blood tests, biopsy and colonoscopic examinations of the intestinal system to obtain a clearer idea of the influence of BT on the state of health of target tissues [52]. It would also be important to investigate in depth, through specific questionnaires and instrumental analyses, how this therapy can correct the psycho-emotional and social aspects related to IBS with CLBP [30]. This is especially true considering how musculoskeletal dysfunctions tend to produce further disorders over time, such as depression and kinesiophobia [53,54]. Moreover, it could be useful to consider advanced instruments capable of objectifying the psycho-physiological state of patients with musculoskeletal and psycho-emotional dysfunctions, which could also compromise their compliance with active and passive therapeutic treatments [55,56,57].

As a final note, we would like to point out that QM is strongly establishing itself in medical specialties [58,59,60], and we await verification of further applications that stand the test of time.

## 5. Conclusions

BT seems to be effective for IBS patients. Given the non-invasiveness, the relatively low cost and the beneficial effects of this treatment, it is strongly suggested to further investigate this approach in patients suffering from IBS and other painful and invasive pathologies. In fact, considering the clinical and bio-psycho-social invasiveness of IBS, it is important to investigate new approaches, especially if they belong to extremely recent medical specialties, as in the case of QM. These investigations will allow us to understand whether these techniques have significant therapeutic properties.

Since the field of instrumental, alternative and complementary therapies is typically of rehabilitative interest, the diffusion of these techniques in the gastroenterology fields is currently limited, with the consequence of slowing down investigations on the effectiveness of these treatments.

It would be desirable to reach an even greater dialog between internal and organ medicine and the rehabilitation field so as to speed up research on innovative therapeutic approaches such as BT or other rehabilitation techniques for the benefit of patients affected by musculoskeletal or organ-related pathologies.

## Figures and Tables

**Table 1:** Specific program designed for IBS for the BT device used in the study.

Internal Program Number	Program Focus	Time
00.00	Analysis Preparation	5 min
01.00	Vitalization Complete	5 min
02.00	Acupuncture Meridians Complete	5 min
31.12	ATP Production Colon	5 min
31.16	ATP Production Small Intestines	5 min
35.10	Raising the Defence Capacity, Basic Program	5 min
70.19	Digestive Organs	10 min
46.00	Digestive System, Physiology Complete	5 min
47.70	Irritable Bowel Syndrome	5 min
75.10	Stress Reduction	5 min
31.50	Basic Detoxification Program	5 min
01.00	Vitalization Complete	5 min

## Data Availability

The data presented in this study, due to the preservation of the privacy of interested parties, could be made available upon motivated and reasonable private request to the corresponding author.

## Supplementary Materials

The following supporting information can be downloaded at: https://www.mdpi.com/article/10.3390/medicina60071099/s1, Table S1: Generalized estimating equations (GEE) estimates (β ± SE), and relative *p*-value for the comparison between times of the study (where follow-up is the reference time) for the items of the SF-36, all the models were adjusted for Calprotectin level.
