# Supplementary material for: Quantum Medicine and Irritable Bowel Syndrome-Associated Chronic Low-Back Pain: A Pilot Observational Study on the Clinical and Bio-Psycho-Social Effects of Bioresonance Therapy
Source: Medicina (Kaunas). 2024 Jul 5;60(7):1099. doi: 10.3390/medicina60071099 (PMC11278534; doi:10.3390/medicina60071099)
Supplement: Supplementary file 1 [file medicina-60-01099-s001.zip › medicina-3033150-supplementary.pdf]

Supplementary material Table S1: Generalized estimating equations (GEE) estimates (β±SE), and relative p-value for the comparison between times of the study (where follow-up is the reference time) for the items of the SF-36, all the models were adjusted for Calprotectin level.

	β±SEβ	p-value
Physical functioning		
Intercept	-0.35±0.07	<0.001
Time		
Baseline	-0.29±0.08	<0.001
Follow-up	reference	
Calprotectin	0.01±0.01	0.86
Physical role limitation		
Intercept	-0.52±0.14	<0.001
Time		
Baseline	-0.65±0.22	0.004
Follow-up	reference	
Calprotectin	0.01±0.01	0.52
Emotional role limitation		
Intercept	-0.16±0.14	0.28
Time		
Baseline	-0.68±0.24	0.006
Follow-up	reference	
Calprotectin	-0.01±0.01	0.24
Vitality		
Intercept	-0.65±0.08	<0.001
Time		
Baseline	-0.51±0.11	<0.001
Follow-up	reference	
Calprotectin	0.002±0.0001	0.01
Emotional wellness		
Intercept	-0.50±0.07	<0.001
Time		
Baseline	-0.37±0.10	<0.001
Follow-up	reference	
Calprotectin	0.01±0.01	0.69
Social functioning		
Intercept	-0.42±0.10	<0.001
Time		
Baseline	-0.30±0.12	0.01
Follow-up	reference	
Calprotectin	-0.01±0.01	0.64
Physical pain		
Intercept	-0.64±0.11	<0.001
Time		
Baseline	-0.64±0.13	<0.001

Follow-up	reference	
Calprotectin	0.003±0.001	0.03
Health		
Intercept	-0.74±0.09	<0.001
Time		
Baseline	-0.39±0.12	0.001
Follow-up	reference	
Calprotectin	0.002±0.001	0.10
